# Theoretical analysis of the cost of antagonistic activity for aquatic bacteria in oligotrophic environments

**DOI:** 10.3389/fmicb.2015.00490

**Published:** 2015-05-27

**Authors:** Eneas Aguirre-von-Wobeser, Luis E. Eguiarte, Valeria Souza, Gloria Soberón-Chávez

**Affiliations:** ^1^Red de Estudios Moleculares Avanzados, Instituto de Ecología A.C.Xalapa, Mexico; ^2^Departamento de Ecología Evolutiva, Instituto de Ecología, Universidad Nacional Autónoma de MéxicoMexico City, Mexico; ^3^Departamento de Biología Molecular y Biotecnología, Instituto de Investigaciones Biomédicas, Universidad Nacional Autónoma de MéxicoMexico City, Mexico

**Keywords:** Bacterial antagonism, diffusion, community ecology, microbial ecology, microbial physiology

## Abstract

Many strains of bacteria produce antagonistic substances that restrain the growth of others, and potentially give them a competitive advantage. These substances are commonly released to the surrounding environment, involving metabolic costs in terms of energy and nutrients. The rate at which these molecules need to be produced to maintain a certain amount of them close to the producing cell before they are diluted into the environment has not been explored so far. To understand the potential cost of production of antagonistic substances in water environments, we used two different theoretical approaches. Using a probabilistic model, we determined the rate at which a cell needs to produce individual molecules in order to keep on average a single molecule in its vicinity at all times. For this minimum protection, a cell would need to invest 3.92 × 10^−22^ kg s^−1^ of organic matter, which is 9 orders of magnitude lower than the estimated expense for growth. Next, we used a continuous model, based on Fick's laws, to explore the production rate needed to sustain minimum inhibitory concentrations around a cell, which would provide much more protection from competitors. In this scenario, cells would need to invest 1.20 × 10^−11^ kg s^−1^, which is 2 orders of magnitude higher than the estimated expense for growth, and thus not sustainable. We hypothesize that the production of antimicrobial compounds by bacteria in aquatic environments lies between these two extremes.

## Introduction

The bacterial production of molecules with antagonistic activity toward other strains is believed to be involved in complex inter-strain interactions at the local scale (Cordero et al., 2012; Pérez-Gutiérrez et al., 2013; Aguirre-von-Wobeser et al., 2014). The role of antagonistic molecules as a means of gaining competitive advantage by killing other cells, has been extensively studied *in silico* (Pagie and Hogeweg, 1999; Czárán et al., 2002; Czárán and Hoekstra, 2003) and in controlled laboratory experiments (Kerr et al., 2002). Alternatively, other types of interactions have been proposed, where these molecules change the behavior of target cells, to elicit biofilm formation, dispersal and other responses (Ratcliff and Denison, 2011). Chemical-mediated competitive interactions can result in local extinction of sensitive strains unless there is a tradeoff paid by the producing strain. An example is the growth penalty resulting from the metabolic cost of the biosynthesis of antagonistic compounds, which results in a disadvantage for the antagonist producing strain in the presence of resistant strains (Kerr et al., 2002; Conlin et al., 2014). If resistant strains pay a metabolic cost for the resistance lower than the cost of being antagonistic, these interactions may result in non-transitive cycles, described as a rock, scissors and paper model (Pagie and Hogeweg, 1999; Frean and Abraham, 2001). If these cycles are stable, permitting that each strain controls another from becoming dominant, coexistence of the strains is favored, while the selective advantage of being antagonistic is maintained. In a different scenario, where an antagonistic strain is able to completely out-compete all sensitive strains, the production of antagonistic mechanisms would soon become disadvantageous (as no competitor strain would survive), and any non-antagonistic (but resistant) mutants could easily be favored by natural selection, since they would not need to spend resources on producing antagonism-molecules in a competitor-free environment.

From the literature exploring rock, scissors and paper models, a consensus view has emerged where a structured environment seems to be necessary for the competitive advantage to result in stable coexistence of resistant, sensitive and antagonistic strains (Durrett and Levin, 1998; Czárán et al., 2002; Kerr et al., 2002; Czárán and Hoekstra, 2003; Greig and Travisano, 2008; Conlin et al., 2014), and therefore, to encourage the stable prevalence of antagonistic compound production. Indeed, antagonistic interactions are extremely common in densely populated, highly structured environments like soils (Vetsigian et al., 2011; Pérez-Gutiérrez et al., 2013) or microbial mats (Long et al., 2013). Surprisingly, bacterial antagonistic activity has also been found in strains isolated from natural water samples with no apparent structure (Lo Giudice et al., 2007; Aguirre-von-Wobeser et al., 2014). Although it is well known that at least some aquatic environments do have structure at the microscopic level (Azam, 1998), they are clearly less stable than sediment environments, and it is not known if that structure is enough to sustain the rock, scissors and paper model dynamics.

Antagonistic mechanisms usually involve the release of molecules to the environment, which can be potentially costly in terms of resources. This poses a paradox for bacterial strains, since each strain needs to invest resources to become more competitive, and this often is needed precisely when resources for growth are scarce. Indeed, higher nutrient availabilities favor antagonistic strains in competition models (Hulot and Huisman, 2004). Bacterial antagonism mechanisms are varied (Konisky, 1982; Michel-Briand and Baysse, 2002; Rebuffat, 2012). In some cases, like pyocins, a single molecule of the antagonistic substance is sufficient to kill a competing cell (Michel-Briand and Baysse, 2002). For instance, one single pore-forming molecule can cause enough depolarization of the membrane of a cell for it to loose viability (Michel-Briand and Baysse, 2002).

One problem that antagonistic strains could face in aquatic environments is the diffusion of antagonistic molecules away from the producing strains. Molecules will wander in random Brownian motion, which will result in the diffusion of the released molecules away from the cell. Therefore, there could be an important cost of producing these molecules, which is expected to depend strongly on the required production rate, according with the target concentration at the vicinity of a cell. In this paper, we propose a model to analyze the order of magnitude of the investment a cell must do to maintain different concentrations of antagonistic molecules. We consider the case where only one molecule of an antagonistic substance in the proximity of the cell is required, as a lower bound of resource allocation, and the case where a certain concentration of these molecules around it is needed, as an upper bound. Based on the results of simulations, we hypothesize that the production of antimicrobial compounds by bacteria in aquatic environments lies between these two extremes. Finally, we put forward the hypothesis that many of these strains regulate the production of antagonistic substances, for example in response to nutrient availability.

## Overview of mathematical approaches

Two approaches were used to establish theoretical boundaries on the costs that cells incur in the production of antagonistic compounds that are released to the environment.

### Probabilistic mathematical modeling

**Figure 1:**
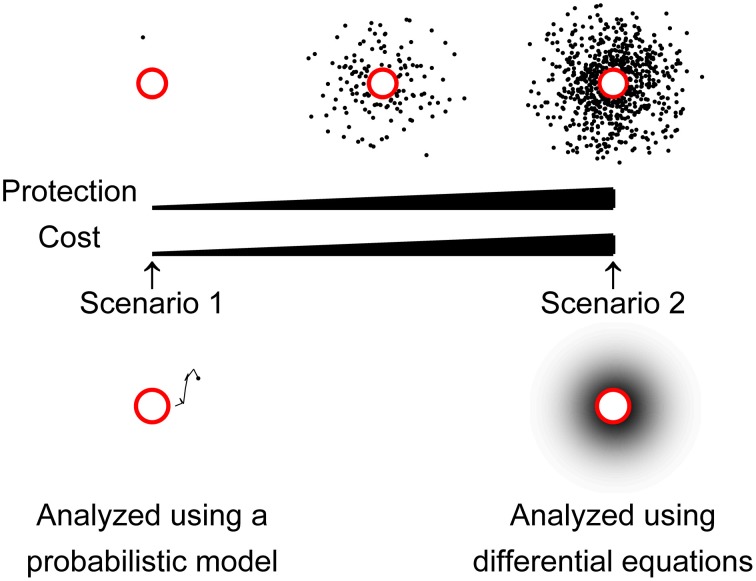
**Schematic illustration of the scenarios considered in this study**. Cells are shown as red circles, and antagonistic molecules as black dots. In the lower left side, an example of a random trajectory of a molecule is represented as a black line. In the lower right side, a gradient represents concentrations of antagonistic molecules, with darker shades indicating higher concentrations. On the left side, a cell keeping on average one molecule of an antagonistic substance in its vicinity is considered. As shown, this would grant the cell an absolute minimum of protection against competing cells, and the associated production cost is considered a lower boundary for resource investment. Note that in these conditions, competing cells still could be close to the producing strain and not encounter the antagonistic molecule. These production costs were calculated using *in silico* simulations in this work, analyzing the random movements of individual molecules, and modeling the distribution of many such simulations (probabilistic modeling). On the right side, another extreme scenario is illustrated, where a cell would obtain full protection by maintaining certain concentration of antagonistic molecules around itself. The ideal concentration was arbitrarily chosen to be the minimum inhibitory concentration, as explained in the text. A cell in an intermediate scenario is also shown for illustration, although it is not explicitly investigated in this work.

In a first scenario, we considered that a cell would need to keep at least one molecule of an antagonistic substance in order to gain a minimum amount of protection from competing cells (Figure 1, left side). With only one molecule on average in the vicinity of a cell, one cannot use mathematical approaches that depend on defined concentrations and gradients, like differential equations. Instead, the problem needs to be approached probabilistically, modeling the fates of individual molecules. After large numbers of simulations, the distribution of the behavior of individual molecules was analyzed using a mathematical model of the distribution, chosen for theoretical reasons, as explained below. We used a probabilistic model based on the Inverse Gaussian Distribution to explore the rate at which a cell would need to produce antagonistic molecules to maintain on average a single molecule in its vicinity at all times.

To simulate diffusion of molecules, simulation experiments were performed to explore the distribution of the time needed for particles to reach a threshold radius, when randomly drifting in Brownian motion. Particles were simulated as if they were released at the surface of the cell, and were allowed to take steps, either toward the cell or away from it. If a particle moved against the cells surface, it was reflected back to its original position. The simulations were performed in one dimension, assuming perfect radial symmetry around an idealized spherical model cell. Five sets of simulations were performed with threshold step-numbers 100, 200, 300, 400, and 500. In each case, 1000 particles were simulated, and the time needed to reach the thresholds was recorded. Inverse Gaussian Distributions were fitted by maximum likelihood using Matlab® (The Mathworks, USA).

### Continuous mathematical modeling

In a second scenario, we considered that a cell would preferably maintain a concentration of antagonistic molecules around itself that could effectively inhibit the growth of potential competitors (Figure 1, right side). For this analysis, since it could be described in terms of concentrations, we used differential equations. For this purpose, we used known solutions to Fick's second law (one of the most important mathematical models of diffusion) to explore the rate at which a cell would need to produce antagonistic molecules, to sustain concentrations that could inhibit the growth of other strains (Ling et al., 2010).

## Mathematical models

### Probabilistic mathematical model to estimate the average time a particle stays close to its producing strain

For the production of antagonistic substances in aquatic environments to be effective, it is reasonable to assume that these molecules need to be close enough to the producing strain to affect cells that are actual potential competitors. We are interested in determining the timescale in which, on average, a molecule produced by a cell is expected to stay close to it, at a small enough distance to potentially interact with competing strains.

What constitutes the vicinity of a cell in terms of competition could be approached in several ways. Cells taking up nutrients from the environment by diffusion create a nutrient concentration gradient around themselves, where the concentration is lowest near the cell surface, and increases monotonically up to the concentration of the environment at a certain radius. Here, we consider two cells of different strains to be competing if the regions of lowered nutrient concentrations overlap. Assuming that nutrients are depleted completely at the cells surface, the concentration of any nutrient around a spherical cell with radius *a* (distances are expressed in meter units throughout this work; for clarity, units are indicated when variables are defined) is simply (Pasciak and Gavis, 1974):

(1) $$C\left(r\right)=C_{\infty }\left(1-\frac{a}{r}\right)$$

expressed as a function of the distance r (radius) from the cells center in any direction, and where C_∞_ (concentrations are expressed in mol L^−1^ throughout this work) is the concentration in the medium. Equation (1) states that the concentration of a nutrient at a given distance from a cell taking it up is a simple function of the radius of the cell and the concentration of the nutrient in the surrounding medium. If we consider the radius where the concentration is 95% of C_∞_ to be the limit where competition is relevant, then a competitor of the same size is in the vicinity if it is at a distance *r* = 2 × 20 = 40 or closer. This distance is the same for any chemical substance taken up by the cell, since Equation (1) does not depend on the diffusion coefficient, or any other property of the chemical involved.

We can assume that an antagonistic molecule released at the surface of a cell will wander randomly through Brownian motion in the medium, due to thermal energy. Random molecular movement has been the subject of extensive theoretical development for almost two centuries, for instance in the context of the study of diffusion. In the traditional view of this phenomenon, the macroscopic flow of molecules (or particles) in a medium with a concentration gradient is thought to be the accumulated result of movements of individual molecules, in steps of nanometer lengths and picosecond timescales, with random directions and mean square velocity v (m/s) given by (Berg, 1993):

(2) $${\langle v^{2}\rangle }^{\frac{1}{2}}={\left(\frac{kT}{m}\right)}^{\frac{1}{2}}$$

Where k is the Boltzman constant (aprox. 1.3806504 × 10^−23^ J K^−1^), T (K) is the absolute temperature and m (kg) is the mass of the molecule or particle. Thus, according to Equation (2), the average velocity of a particle depends on the temperature and on its mass.

The discrete nature of these molecular movements is thought to reflect the trajectory between collisions with one molecule of the surrounding medium to the next one; trajectories whose average length is known as the mean free path. According to this random walk model of diffusion, the individual solute molecules very rarely meet (given sufficiently low concentrations), and their trajectories and displacements are therefore considered independent. This assumption of traditional diffusion theory allows us to utilize the exact same theoretical framework to study the mean displacements of individual molecules released at different times at a cells surface.

**Figure 2:**
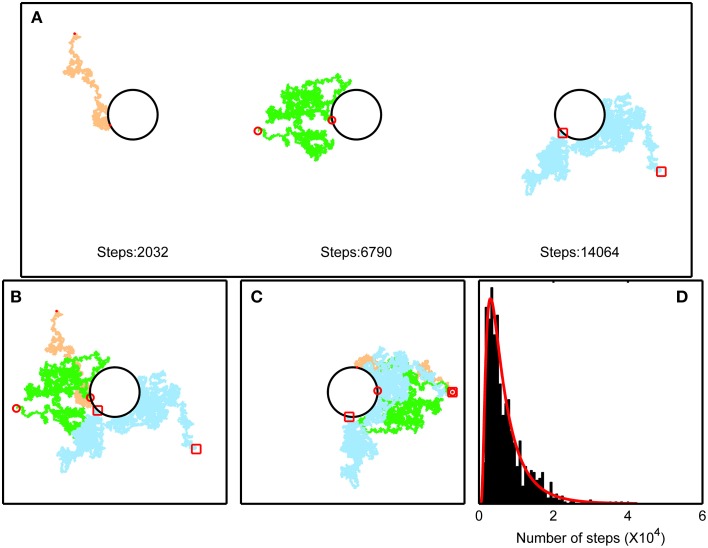
**Simulation to illustrate “random walk” movements of a particle away from a cell (black circles) that produced it. (A)** Trajectories of three different particles produced by the same cell at different time points are shown as colored continuous lines (different colors are used to distinguish the three independent particles; red dots, circles and squares mark the start and end positions of the three independent particles), since the time they are released, until they reach a distance of 3 cell radiuses from the cell surface for the first time. Notice that they will approach the cell again with probability 0.5 at the next step, or drift further away. The number of steps taken to reach that position, is indicated for each case. Note that the length of each step is greatly exaggerated in this figure, as compared to the scales of real molecules and bacteria. **(B)** The trajectories are plotted around the same depiction of the cell, putting them in the same time-frame. **(C)** The trajectories are rotated, putting them in the same spatial frame, illustrating how these bi-dimensional trajectories can be studied in one dimension if the only quantity of interest is the distance from the cell. This same reasoning can be applied for a three-dimensional scenario. **(D)** Distribution of the number of steps taken to reach 3 cell radiuses in 1000 simulations with the same scales as in panel **(A)**. The red line indicates an Inverse Gaussian Distribution fitted to the data. Note that an equally well fit can be attained using a Birnbaum-Saunders Distribution, but the Inverse Gaussian is preferred for theoretical reasons.

Due to the random distribution of the solvent molecules, in any frame of reference in a three dimensional environment, the displacement of diffusing particles is known to be independent for each of the three spatial dimensions (Berg, 1993). For convenience, we set the frame of reference arbitrarily parallel to the maximum displacement of each particle when they reach radius *r* (Figures 2A–C). Note that this is similar to setting the time frame of reference for every particle at the time they were excreted at the surface of the cell. Thus, we can model the dispersion of particles as a one-dimensional random walk (Crank, 1975). In other words, since the components of random movements that are not normal (perpendicular) to the cells surface in the direction that randomly generates the maximum displacement, do not contribute to drive the molecules away from the cells, only normal displacement in that direction need to be modeled (note that we only need to model one dimension, along the radius, although the particles actually move in three dimensions). Since we assume that the molecules are not absorbed again at the cells surface, we consider it a reflected random walk, as the molecules are reflected when they collide with the cells surface. With sufficiently large numbers of particles, the probability of finding a particle at a distance *r* from the origin at time *t* follows a normal distribution (Berg, 1993):

(3) $$p\left(r,t\right)=\frac{1}{\sqrt{4\pi Dt}}e^{-\frac{r^{2}}{4Dt}}$$

where *D* (m^2^ s^−1^; note that, to use a single unit system, we do not follow the common practice of expressing *D* as cm^2^ s^−1^) is the diffusion coefficient, and the variance is 2*Dt*. So, the expected number of particles will decline with the distance from the cell, and the variations around this expected number will be greater with higher diffusion coefficients.

**Figure 3:**
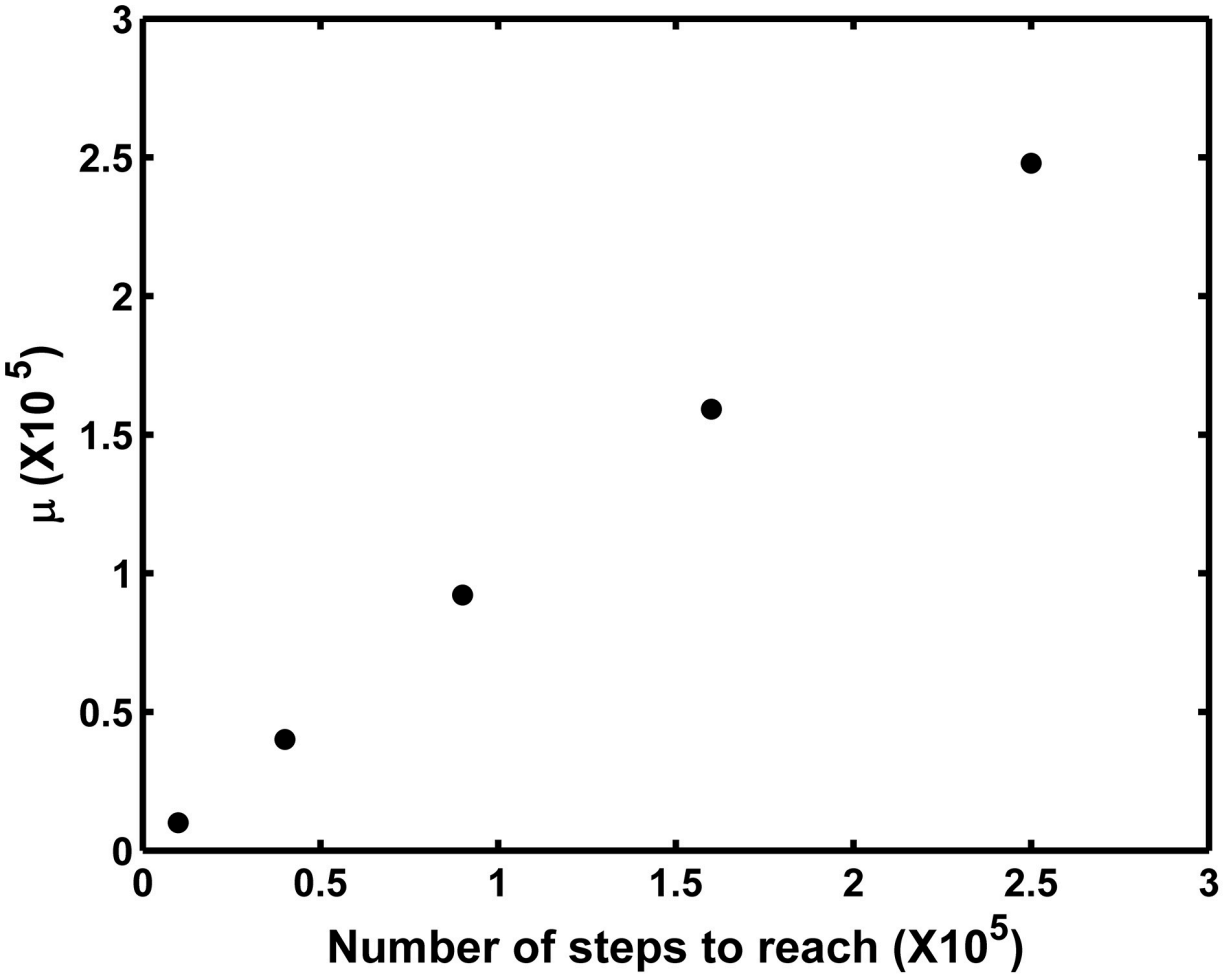
**Simulation experiment showing the relation between parameter μ of the Inverse Gaussian Distribution, and the square of the number of steps n^2^_b_ needed to reach a radius n_b_ from the surface of the cell, measured in step-lengths**. For each point, 1000 particles were allowed to wander with steps of fixed length and random direction, simulating Brownian motion, until they reached the radius indicated in the x axis, and the number of steps taken were recorded. The values correspond to parameter μ, obtained by fitting the Inverse Gaussian Distribution to the corresponding 1000 times. We notice that this relation can most likely be obtained analytically, but do not count with a mathematical proof at the moment.

To model the time needed for a collection of molecules released at the cells surface to reach a threshold distance from the cell (say 40 cell radiuses), we can consider each step to be a normally distributed random variable, as described by Equation (3). The distribution of the number of steps needed by particles to reach a threshold distance *r* = b can be modeled statistically under certain premises. In the case of a random walk with positive drift (for example when a current is present) it can be described by an Inverse Gaussian Distribution, which has a probability density function (Folks and Chhikara, 1978):

(4) $$p\left(t,\mu ,\lambda \right)=\left(\frac{\lambda }{2\pi t^{3}}\right)e^{\frac{-\lambda {\left(t-\mu \right)}^{2}}{2\mu^{2}t}}$$

for *t* > 0, where μ is the mean number of steps needed to reach distance b, and λ is a shape parameter which, together with the mean, defines the variance as μ^3^/λ. The time to reach a threshold radius in a reflected random walk has also an Inverse Gaussian Distribution (Figure 2D), even when no drift is present (Abate and Whitt, 2011). Note that the problem is described mathematically as a one-dimensional, focusing on the relevant dimension, being the distance of the particle from the cell, as explained above. A simple simulation study shows that μ is equal to n^2^_b_ where n_b_ is the number of steps needed to reach b (Figure 3). Although we lack mathematical proof of this empirically observed relation, it allows us to express the average number of steps needed to reach arbitrary radius b as a function of useful quantities, as follows. If each step takes an average τ s, the mean time to reach b is 〈t_b_ 〉 = τ n^2^_b_, that is, the average number of steps times the average time they take. From the definition of velocity as *v* = δ/τ, and the definition of the diffusion coefficient as *D* = δ^2^/2τ, one can express τ in terms of known quantities as:

(5) $$\tau =\frac{2D}{v^{2}}=\frac{2Dm}{kT}$$

which gives us a simple expression for the average step time, and we can express the average step size as:

(6) $$\delta =\tau v=\tau {\left(\frac{kT}{m}\right)}^{\frac{1}{2}}=\frac{2Dm}{kT}{\left(\frac{kT}{m}\right)}^{\frac{1}{2}}=2D{\left(\frac{m}{kT}\right)}^{\frac{1}{2}}$$

The number of steps to reach b is equal to n_b_ = b/δ which, with *b* = 40a, equals n_b_ = 40a/δ. Thus:

(7) $$\langle \text{tb}\rangle =\tau n_{b}{}^{2}=\tau \;1600\frac{a^{2}}{\delta^{2}}=\frac{2Dm}{kT}1600{\text{a}}^{2}\frac{1}{4D^{2}}\frac{kT}{m}=800\frac{a^{2}}{D}$$

Using Equation (7), we can calculate the average time for molecules to reach a distance of 40 cell radiuses from the cell, by knowing their diffusion coefficients. Diffusion coefficients for small molecules have an order of magnitude of 10^−9^ m^2^s^−1^ while for small proteins they tend to range from 10^−10^ to 10^−11^ m^2^s^−1^ (Erickson, 2009). For example, for an s5-type pyocin from *Pseudomonas aeruginosa* which can target other strains from the same species (Ling et al., 2010), we can calculate the diffusion coefficient using the Einstein-Stokes relation:

(8) $$D=\frac{kT}{6\pi \eta r}$$

where η is the viscosity, which for water at 291.15 K (18°C) is about 0.001009 Pa s, and the radius of the protein can be estimated (Erickson, 2009):

(9) $$r=6.6\times \;10^{-11}M^{\frac{1}{3}}$$

where *M* is the molecular weight *M*, which for this protein is 57.6 kDa (Ling et al., 2010). Equation (8) gives an estimated diffusion coefficient of *D* = 8.29 × 10^−10^. For a cell with radius *a* = 0.5 × 10^−6^m (0.5 μm), Equation (7) estimates the average time for a molecule of the said pyocin to stay close to the producing cell to be about 0.24 s.

Thus, in order to maintain at least one molecule of an antagonism molecule, of similar size, at a distance close enough to be able to affect a competitor, a cell would need to produce them at a rate of 4.1 molecules s^−1^, or 6.88 × 10^−24^ mol s^−1^. We regard this figure as the minimum investment that would make any sense in an aquatic environment (Figure 1, left side). However, in order to be really protected, and gain competitive advantage, a cell would need to maintain a concentration of antagonism molecules around itself (Figure 1, right side). Therefore, we are also interested in the rate of synthesis that a cell would need to sustain in order to keep certain concentration of antagonism molecules around it.

### Fick's laws based model to determine the production rate to sustain a minimum inhibitory concentration around a cell

In the second approach, we considered the cost for the cell to maintain a certain concentration around itself to inhibit potential competitors. For this approach, to model individual molecules becomes impractical, but the continuous gradients provided by large numbers of molecules allow us to use differential equations of known diffusion theory (Figure 1, right side). The change in concentration *C* of a substance at a distance *r* in the presence of a gradient can be described by Fick's second law, which around a sphere has the following form (Crank, 1975; Berg, 1993):

(10) $$\frac{\partial C}{\partial t}=\frac{D}{r^{2}}\frac{\partial }{\partial r}\left(r^{2}\frac{\partial C}{\partial r}\right)$$

Equation (10) is only valid when the number of molecules produced is high enough to be adequately described as a continuum concentration. There is no general solution (lacking unknown integration constants) for Equation (10), but solutions depending on particular conditions of a given setting can often be found. These conditions are termed boundary conditions, and aid in determining the values of integration constants to obtain particular solutions for the problem in hand. For instance, if a spherical cell is producing a substance and releasing it a constant rate at the surface, Equation (10) can be solved assuming a steady state with boundary conditions *C*_∞_ = 0 (no antagonistic molecules exist in the surrounding medium, and a current *I* (mol s^−1^) originating at the cells surface (the production of the molecules by the cell), where *C*_∞_ is the concentration of the molecule in the medium away from the cell. This problem has known solution:

(11) $$C\left(r\right)=\frac{I}{4\pi Dr}$$

Equation (11) conveniently expresses the concentration of the antagonistic molecules as a function of the distance from the cell, according the constant production rate and the diffusion coefficient. Since the concentration is lowest at the outer extreme of the region of interest, by taking *r* = 40*a*, we make sure that the concentration is high enough in the whole region between this radius and the surface of the cell:

(12) $$C_{r=40a}=\;\frac{I}{160\pi Da}$$

Now we need a value for the ideal concentration in the interest region. For this purpose, we use the concept of minimum inhibitory concentration (*MIC*), which is the minimum concentration that yields a noticeable decline in growth of sensitive strains. This minimum concentration required to inhibit a potential competitor depends on the specific antimicrobial substance and the particular sensitive strain. Studies can be found that measure this quantity (e.g., Ling et al., 2010) and report empirically determined *MIC* values. Using this concept, we substitute *C*_*r*=40*a*_ for *MIC* and get:

(13) I=MIC 160πDa

Values of *MIC* are typically in the order of the tens of microgram per milliliter. For example, for the s-type pyocin mentioned in the preceding section, a measured *MIC* of 0.0126 kg/m^3^ against a specific target *P. aeruginosa* strain has been reported (Ling et al., 2010). Using the diffusion coefficient calculated with Equation (8), Equation (13) gives a value of *I* = 2.1 × 10^−13^ mol s^−1^, or 1.26 × 10^11^ molecules per s.

### Cost of producing and releasing an antagonistic substance

Under the assumption that antagonism molecules have the same elementary composition as the average stoichiometry of the cell, the production and release of those molecules would be limited by the same nutrient(s) as growth. So, comparing the mass range of these molecules and the mass and growth rates of typical bacteria, we can estimate if the production rates we calculated in the previous sections are sustainable.

To obtain the dry weight of a spherical bacterium, we can use an empirical relation between the cells volume and the dry weight *W* (kg cell^−1^), valid for cells larger than 0.025 μm^3^ (Romanova and Sazhin, 2010):

(14) $$W=133.745\;\times \;10^{-12}V^{0.438}=133.745\;\times \;10^{-12}{\left(\frac{4}{3}\pi a^{3}\right)}^{0.438}$$

For example, for a cell with radius a = 0.5 × 10^−6^m, the dry weight would be approximately 1.32 × 10^−18^ kg. To sustain this biomass, at a growth rate of 1 division per day (1.16 × 10^−5^ s^−1^), the cell would need to produce biomass at a rate of 1.14 × 10^−13^ kg s^−1^. For the pyocin discussed above (Ling et al., 2010), the mass of a single molecule would be 9.56 × 10^−23^ kg. It was determined that a cell would need to produce molecules at a rate of 4.1 s^−1^ to have, on average, a single molecule of an antagonistic compound in its vicinity at all times (Scenario 1), which amounts to 3.92 × 10^−22^ kg s^−1^. Compared to the cost of biomass production for growth, this is about 9 orders of magnitude lower. However, to produce enough molecules to sustain a minimum inhibitory concentration, a cell would need to produce molecules at a rate of 1.26 × 10^11^ s^−1^, or 1.20 × 10^−11^ kg s^−1^. This value is two orders of magnitude larger than the cost of biomass production.

## Discussion

Many bacterial species produce substances to hamper the growth and proliferation of others in densely populated or highly structured environments (Vetsigian et al., 2011; Long et al., 2013; Pérez-Gutiérrez et al., 2013). We are concerned with the theoretical challenge posed by the presence of antagonistic mechanisms in water environments as well (Long and Azam, 2001; Lo Giudice et al., 2007; Aguirre-von-Wobeser et al., 2014). Moreover, these mechanisms can be found in highly oligotrophic environments like the Churince system in Cuatro Ciénegas (Aguirre-von-Wobeser et al., 2014) where there is not only low structure, but the cell densities are much lower, and the probability of encounter with other cells is very low, and where molecules can easily diffuse away from the producing strain. We specifically addressed this last issue, since we were interested in calculating the order of magnitude of the frequency of production of antagonism molecules that would make sense for a cell to sustain. We considered two different scenarios:

1. A cell that would only need to keep a single molecule of an antagonistic substance in its close proximity most of the time. This scenario is supported by the observation that for some antagonistic compounds, a single molecule can kill a competing strain (Michel-Briand and Baysse, 2002).
2. A cell that would need to keep a minimum inhibitory concentration around itself.

Since the two scenarios are best described by discrete and continuous mathematical models, respectively, we used different theoretical approaches to further our understanding of the cost of antagonism in a water environment. First we analyzed the situation where a single molecule around a producing strain could be advantageous. In this scenario, where a minimum production of antagonistic molecules is expected, we found the cost of production in terms of biomass to be nine orders of magnitude lower than the metabolic cost of net growth. However, with such a low release of antagonistic substances, the cell would need to rely on the chance encounter of the antagonistic molecule with the target strain, which could be highly random. Therefore, we regard this absolute minimum antagonism molecule production as not likely to be enough for an effective protection from competitors.

Then we analyzed the cost of sustained production of antagonistic molecules to create a minimum inhibitory concentration around the producing strain. To achieve this, the cell would need to sustain a constant production of antagonistic molecules in high enough quantities to replenish all the molecules that constantly diffuse away. As expected, the calculated cost contrasted sharply with what was found in the previous case. The rate of molecule production the cell would need to sustain turned out to be even higher than the cost of growth by net biomass synthesis, by two orders of magnitude, when considering one doubling event per day. This means that the cell would need to spend much more resources for antagonism molecule production than for growth. In real life, it is not likely that the cells invest continually these high amounts of resources. For example, *Escherichia coli* cells carrying different colicin-producing plasmids show a growth reduction of approximately 20% (Gordon and Riley, 1999).

The models used in this study were developed to understand antagonistic substance production in aquatic oligotrophic environments with low, possibly negligible structure. However, they could be adapted to scenarios where structure is more prominent, such as soils, root-associated communities, snow environments, eutrophic aquatic environments, marine-snow rich oceanic waters, among others, to understand the role of diffusion in such environments.

Although it is possible that the cells synthesize antagonistic compounds at an intermediate rate between the two extremes considered, another likely scenario is that these mechanisms are regulated by differential gene expression, and are only turned on under certain conditions. Bacteria are known to be very flexible in their gene expression profiles in varying environmental conditions (e.g., Aguirre-von-Wobeser et al., 2011; Dugar et al., 2013). Modulation of the production of antagonistic substances in response to environmental conditions has been observed (Bruhn et al., 2007). A recent study has explored regulation of colicin gene expression in *Escherichia coli*, and found a very dynamic regulation of antagonism (Hol et al., 2014). Therefore, we hypothesize that regulation of antagonistic molecule synthesis is an important strategy for cells to produce these compounds in the environment when they are cost-effective.

It is a highly complex question to establish at which concentrations of nutrients it makes sense for a bacterium to produce antagonistic substances, and it falls outside the scope of this article. However, to have some insight on this problem, the costs of production of these molecules for aquatic bacteria calculated in this work could be inserted in growth models that consider maintenance costs, like those discussed in van Bodegom (2007). Additionally, such studies would need to include several experimentally obtainable parameters, like growth kinetic parameters of producing and sensible strains and growth penalties for competing strains with different concentrations of antagonistic substances.

A possibility that cannot be ruled-out is that at least some cells have proteins in their external surface that could reversibly attach to antagonism molecules. In that case, the times in which the molecules would stay in the cells vicinity could greatly increase. To the best of our knowledge, this question remains unanswered.

We conclude that by using theoretical approaches and observations from the literature, we found that the production of antagonism by bacteria in non-structured environments is a complex phenomenon that is likely to be highly regulated. Nutrient availability, cost of production of molecules with antagonistic activity, diffusion of these molecules and cell motility are likely forces participating in the process.

### Conflict of interest statement

The authors declare that the research was conducted in the absence of any commercial or financial relationships that could be construed as a potential conflict of interest.
